# Muscle Strength and Neuromuscular Control in Low-Back Pain: Elite Athletes Versus General Population

**DOI:** 10.3389/fnins.2018.00436

**Published:** 2018-07-03

**Authors:** María Moreno Catalá, Arno Schroll, Gunnar Laube, Adamantios Arampatzis

**Affiliations:** ^1^Department of Training and Movement Sciences, Humboldt-Universität zu Berlin, Berlin, Germany; ^2^Berlin School of Movement Science, Berlin, Germany

**Keywords:** trunk muscle strength, local dynamic stability, quick-release, onset time, erector spinae, MiSpEx

## Abstract

The purpose of the study was to investigate the athletic-based specificity of muscle strength and neuromuscular control of spine stability in chronic non-specific low-back pain (LBP). Thirty elite athletes and 29 age-matched non-athletes with (15 athletes and 15 non-athletes) and without LBP (15 athletes and 14 non-athletes) participated in the study. Muscle strength was measured during maximal isometric trunk flexion and trunk extension contractions. The neuromuscular control of spine stability was analyzed by determining trunk stiffness, trunk damping, and onset times of the lumbar and thoracic erector spinae muscles after sudden perturbations (quick release experiments) as well as maximum Lyapunov exponents (local dynamic stability) using non-linear time series analysis of repetitive lifting movements. LBP was assessed using the visual analog scale. We found lower maximal trunk extension moments (*p* = 0.03), higher trunk damping (*p* = 0.018) and shorter onset times (*p* = 0.03) of the investigated trunk muscles in LBP patients in both athletes and non-athletes. Trunk stiffness and the local dynamic stability did not show any differences (*p* = 0.136 and *p* = 0.375, respectively) between LBP patients and healthy controls in both groups. It can be concluded that, despite the high-level of training in athletes, both athletes and non-athletes with LBP showed the same deconditioning of the lumbar extensor muscles and developed similar strategies to ensure spine stability after sudden perturbations to protect the spine from pain and damage. The findings highlight that specific training interventions for the trunk muscles are not only crucial for individuals of the general population, but also for well-trained athletes.

## Introduction

Low-back pain (LBP) is a worldwide-recognized problem that has become one of the major issues for public health systems with dramatic consequences for the quality of life of the affected patients (Deyo et al., 2006; Louw et al., 2007). It affects up to 84% of the general population in a lifetime (i.e., ranging from 51 to 84%; Manchikanti et al., 2009; Taylor et al., 2014), having a great impact on health care cost (Hammill et al., 2008; Balagué et al., 2012). Between 24 and 80% of the patients report having experienced recurrent LBP and 8% of the total population of patients develop a chronic pathology (Hoy et al., 2010; Reid et al., 2011). The great majority of the LBP patients is diagnosed with “non-specific” LBP, since the diagnosis of a recognizable, specific pathology is missing (van Tulder et al., 2006). LBP affects not only individuals of the general population but also specific subgroups like elite athletes. Epidemiological studies report very high lifetime prevalence, which is depending on the sport discipline (Schulz et al., 2016). Prevalence rates of up to 66% have been reported for cross-country skiers (Eriksson et al., 1996) and even of 94% for rowers (Ng et al., 2014). Non-specific LBP can have dramatic consequences on athletic performance, ranging from chronic injuries to absenteeism from training and competition (Mortazavi et al., 2015). Indeed, pain of the lower back has been shown to be one of the most common reasons among male professional football players for missed playing time (Bono, 2004).

There are multiple risk factors predisposing an individual to develop LBP and their interaction is extremely complex (Cholewicki et al., 2005). Alterations in muscular activity patterns (Van Dieën et al., 2003), reductions in muscle size and strength (Ebenbichler et al., 2001; Beneck and Kulig, 2012), and impaired neuromuscular control of spine stability (Cholewicki et al., 2005) have been associated with LBP in the general non-athletic population. For example, impaired motor control of the trunk after sudden, unexpected perturbations has been related to an increased risk of low-back injury in a prospective experimental design (Cholewicki et al., 2005). Furthermore, previous studies reported reduced motor control in LBP patients during repetitive dynamic trunk motion compared to healthy controls (Graham et al., 2014; Ross et al., 2015). Using non-linear time series analysis, the last-mentioned studies found higher maximum Lyapunov exponents in LBP patients, indicating an increased occurrence of motor control errors in the presence of LBP. Therefore, exercise interventions aiming to improve muscle strength and neuromuscular coordination have been generally accepted as one of the most effective treatments to prevent and to reduce LBP in the general population (Jeffries et al., 2007; Bigos et al., 2009; Choi et al., 2010).

In athletes, high intensity strength training and highly demanding coordinative exercises are the daily basis of their training routine. From this point of view, it could be expected that this high level of practice would produce specific adaptations in the trunk muscles and the neuromuscular control of the spine in athletes. For example, pain perception and pain modulation in athletes can be different compared to normally active controls (Tesarz et al., 2012, 2013). Recently, it has been reported that athletes show significantly lower psychosocial risk profiles and prognostic risks compared to non-athletes (Wippert et al., 2017). Although both muscle strength and neuromuscular control of trunk stability have been often investigated in non-athletes, knowledge regarding these properties in elite athletes with and without non-specific LBP is limited (Trompeter et al., 2017). It is unclear if a deterioration in muscle strength and in the neuromuscular control of trunk stability is associated with LBP in this population. Especially in elite athletes, where a high training volume and training intensity is required, information concerning these two risk factors and their association to LBP can be important for the development of effective treatment strategies to reduce the occurrence of LBP in the athletic practice.

The purpose of the current study was to investigate the athletic-based specificity of muscle strength and neuromuscular control of spine stability in chronic non-specific LBP, performing a systematic comparison between athletes and non-athletes with and without non-specific LBP. We expected to find specific pathology-related effects in athletes, different to those in non-athletes. Muscle strength exercise is a main component of the athletic training practice (Kraemer et al., 1998) and, therefore, we hypothesized that LBP in athletes will not be associated to a reduction in muscle strength. However, a direct transfer of muscle strength to an efficient motor control of the spine in response to sudden unexpected perturbations and/or control errors, which actually can be initiated from deficits in the perception and processing of sensory information, is questionable (Steele et al., 2015). Therefore, we hypothesized an association of the deterioration in neuromuscular control of spine stability with LBP in athletes.

## Materials and Methods

### Participants

A total sample of 59 volunteers (39 men and 20 women) between 19 and 31 years of age participated in this study, being either elite athletes (“athletes”; *n* = 30) or individuals from the general population (“non-athletes”; *n* = 29) and either with (“LBP”) or without (“healthy”) chronic non-specific LBP (**Table 1**). The group of non-athletes practiced diverse sport activities (e.g., jogging, swimming, and cycling) at a recreational level (average hours of regular sport activity per week of 3.2 ± 1.9 and 3.5 ± 3.8 for the healthy and the LBP groups, respectively). Only two participants in the LBP group and another two participants in the healthy non-athlete group did not practice any sport activity at all. The group of athletes involved participants of different sports [soccer, handball, judo, gymnastics, and athletics (discus and javelin throwing)] that trained at least four times a week (average hours per week of 10.4 ± 2.7 and 11.4 ± 1.9 for the healthy and the LBP athletes, respectively) and participated regularly in national or international competitions. The inclusion criteria for the LBP participants were (a) having experienced chronic non-specific back pain (i.e., pain not attributable to a recognizable, known specific pathology) within the last 12 weeks and (b) evidence of LBP induced limitations during daily activities. Exclusion criteria included any previous history of spinal operation, prolapse, herniated disks, arthritis, mental, neurological, or cardiovascular diseases, sensorimotor deficits, abnormal spinal column structural changes, continuous dependency of pain relieve medication, restrictions from participating in sporting activity from a doctor or undergoing physiotherapist treatment. Healthy controls had not experienced lower back pain within the last 12 weeks. Participants signed informed consent for participation within this study, which was approved by the ethics committee of the Charité (“Universitätsmedizin Berlin”).

**Table 1:** Anthropometric data, visual analog scale score (VAS) and regular sport activity in hours per week (Sport) of the four investigated groups.

Group	Age (years)	Mass (kg)	Height (m)	BMI (kg/m^2^)	VAS (cm)	Sport (h/week)
Healthy non-athletes (*n* = 14; ♀ 5, ♂ 9)	24 ± 3	70.3 ± 11.2	1.75 ± 0.11	22.7 ± 2.5	0 ± 0	3.2 ± 1.9
LBP non-athletes (*n* = 15; ♀ 5, ♂ 10)	27 ± 1	78.0 ± 17.7	1.78 ± 0.07	24.3 ± 4.3	3.92 ± 1.70	3.5 ± 3.8
Healthy athletes (*n* = 15; ♀ 5, ♂ 10)	23 ± 3	73.4 ± 13.4	1.78 ± 0.09	22.8 ± 2.4	0 ± 0	10.4 ± 2.7
LBP athletes (*n* = 15; ♀ 5, ♂ 10)	23 ± 2	72.9 ± 10.7	1.75 ± 0.10	23.6 ± 1.6	4.54 ± 1.82	11.4 ± 1.9
*p*-value pain effect	0.054	0.315	0.909	0.123	0.000	0.410
*p*-value group effect	0.002	0.789	0.925	0.724	0.353	0.000
*p*-value interaction	0.094	0.253	0.227	0.587	0.353	0.639

*Elite athletes (athletes), with (LBP), and without (healthy) chronic non-specific low-back pain and participants of the general population (non-athletes) with and without chronic non-specific low-back pain (means ± standard deviation and p-values). BMI, body mass index.*

### Muscle Strength Assessment

Trunk muscle strength was measured during maximal isometric contractions using a dynamometer (Biodex 3 Medical System Inc., United States) with a dual position back extension/flexion seat attachment. The participants were seated on the adjustable seat, fastened with velcro straps over the torso, hip, and thigh to isolate the trunk movement. The axis of the dynamometer was aligned with the subject’s L5/S1 disk space (Granito et al., 2012).

After an initial warm up consisting of several submaximal and two to three maximal contractions, the participants performed maximal isometric trunk extension and flexion contractions at three different trunk positions (-15°, 5° and 30° trunk angle). Zero-degree trunk angle corresponded to the neutral-seated position (i.e., trunk perpendicular to the thighs) with negative values in extended and positive values in flexed trunk position. At every position, the participants completed one trial in extension and one in flexion in order to avoid the appearance of fatigue during the test. All contractions were performed in a randomized order and, during the experiment, the participants were verbally motivated to ensure maximal effort. The participants were interviewed about their perception of pain and effort during the maximal voluntary contractions to exclude any acute pain effects on the muscle strength measurements. In all our measurements, participants did not mention any pain during any of the trials. Three minutes of rest was allowed between the contractions. For the analysis, moment values were normalized to body mass.

### Trunk Stability Assessment

Neuromuscular control of spine stability was analyzed by determining the trunk instantaneous stiffness and damping after sudden perturbations as well as the local dynamic stability during repetitive trunk movement.

#### Stiffness and Damping Coefficients

**FIGURE 1:**
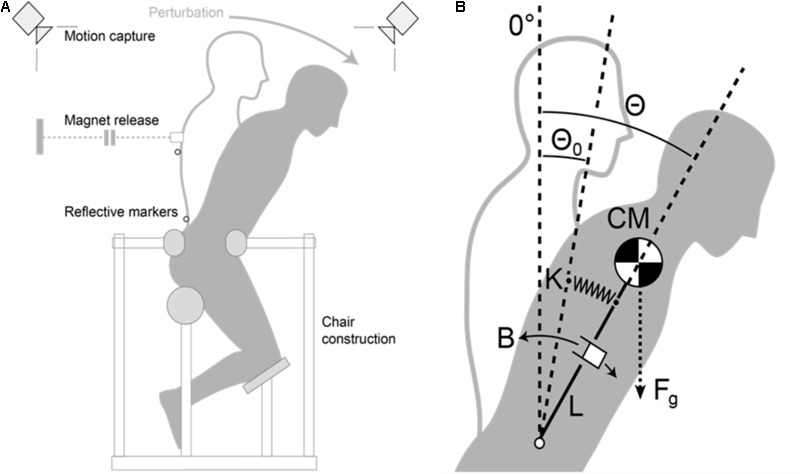
**(A)** Schematic figure of the custom-developed perturbation-system for the quick release experiment. **(B)** Spring model of the trunk with stiffness *K*, damping *B*, resting angle θ_0_ (hypothetical resting angle of spring stiffness), trunk length *L* (measured from the L4/L5 to the T9 joints), gravitational trunk force F_g_, and instantaneous angle of the trunk θ(*t*).

Instantaneous trunk stiffness and damping coefficients were calculated from the kinematic data of the trunk response during a quick release experiment, in which during an isometric trunk flexion a sudden unexpected in time unloading (quick release) was generated by a custom-developed perturbation-system (Cholewicki et al., 2000; Radebold et al., 2000). After a general warm up of the trunk muscles, the participants were placed in a semi-seated position in a specially built apparatus, which was designed to restrict pelvis movement with anchoring cushions placed at the rear and front of the pelvis, allowing the trunk to move free in all directions (**Figure 1**). By restricting the movement of the pelvis, we excluded any postural adjustment through joints inferior to the spine (i.e., hip, knee, and ankle). The semi-seated position allowed the participants to assume their most comfortable upright lumbar spine position before the pelvis was restrained. A cable was then attached to a chest harness at T9 (ninth thoracic vertebrae) height and was held with an electromagnet (Tremba GmbH, Germany, ≥2.16 kN). A force sensor (MEGATRON Elektronik GmbH & Co. KG, Germany, 0–5 kN, 2073 Hz) was placed along the cable between the chest harness and the electromagnet to measure trunk flexion force exertion during the experiment.

After a second warm up exercise consisting of several submaximal trunk flexion and extension contractions, two maximal voluntary isometric flexion contractions (MVC) were performed in the upright position. After the MVCs, the participants performed five quick release trials. In every trial, the participants pulled against the electromagnet until they exerted an abdominal bracing force of 35% of their highest MVC while remaining in a neutral upright position. Once the participants reached 35% MVC, the release was randomly triggered. The participants were instructed to decelerate trunk motion, to regain balance, and to return to the upright position as fast as possible once released.

Kinematic data of two anatomical points (L4/L5 disk space as axis of rotation and ninth thoracic vertebrae as the trunk’s center of mass) as well as the force signal were recorded simultaneously using a Vicon 624 system (Vicon Motion Systems, United Kingdom, 250 Hz) to analyze the trunk response and to ensure that participants maintained an accurate upright position during all test trials. The trunk was modeled as a damped spring (represented by a second-order linear differential equations system), oscillating freely after the release of the resisted torque (35% MVC). Thus, trunk rotation angle is determined by the trunk inertia *I*, damping coefficient *B*, stiffness coefficient *K*, resting angle θ_0_, segment mass *m*, and trunk length *L* (L4/L5-T9; Cholewicki et al., 2000):

(1) $$I\cdot \theta^{\prime \prime }\left(t\right)+B\cdot \theta^{\prime }\left(t\right)+K\left(\theta \left(t\right)-\theta_{0}\right)=m\cdot g\cdot L\cdot \sin\left(\theta \left(t\right)\right)$$

the trunk moment of inertia and the segments’ mass were calculated based on the data reported by Winter (1990). Stiffness and damping were estimated with a curve-fitting algorithm, which calculated the best match between the modeled and the measured trunk rotation kinematic data from release until maximum trunk flexion angle. The average values of *B* and *K* from the five trials normalized to body mass were used for the statistical analysis.

Bipolar EMG leadoffs (Biovision GmbH, Germany) with pre-amplification (bandwidth 10–500 Hz) were used to measure the electromyographic activity of the lumbar and thoracic erector spinae. Differential, circular shaped electrodes with Ag/AgCl-sensors (Ambu BlueSensor N) were placed with an interelectrode distance of 2 cm on the left side of the body (3 cm lateral to the fourth/fifth lumbar vertebrae and 3 cm lateral to the ninth thoracic vertebrae) after cleaning and shaving the skin. Since we used the EMG recordings only for the assessment of the onset time of the erector spinae muscles, we restricted the recording to one side of the trunk assuming similar onset times of both sides. The EMG signals were recorded with the Vicon system at 2073 Hz sampling frequency. A median filter with a window width of 26 data points was applied to the full-wave rectified EMG signal. The onset times of both erector spinae muscles were defined as the time between release and the instant at which the filtered signal exceeded the mean plus three standard deviations of the resting EMG-signal. The EMG signal at rest was taken from the filtered signal in the interval 200 to 50 ms prior to the release (Morey-Klapsing et al., 2004). The mean value of five consecutive trials was used for the statistical analysis in order to better estimate the average onset times of each participant and to reduce possible methodological bias.

#### Maximum Lyapunov Exponent

**FIGURE 2:**
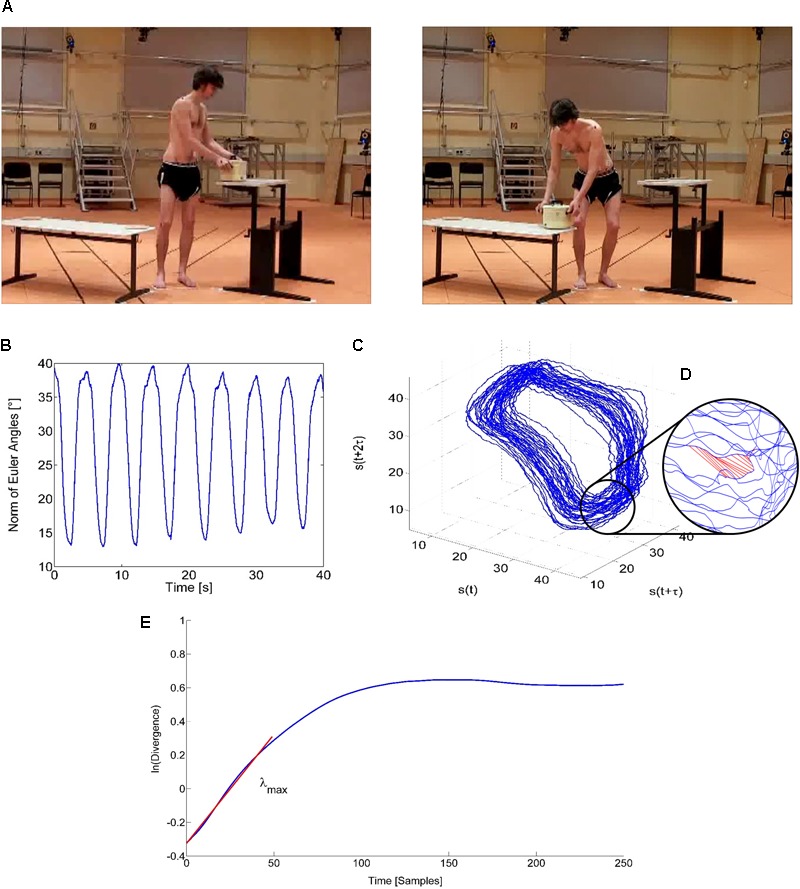
Assessment of the local dynamic stability of the trunk during the lifting task. **(A)** The participants repeatedly executed a lifting task from the left to the right table and back with a frequency of 12 cycles per minute (0.2 Hz) holding a pot with a mass of 1.5 kg. Written informed consent was obtained from the individual for the publication of this image. **(B)** Non-linear time series analysis. Instantaneous data of the Euclidean norm of the trunk’s Euler angles. **(C)** Reconstructed state space of the spine dynamics during lifting motion using dimension *m* = 3 and time delay *τ* = 1.2 s. **(D)** Diverging Euclidean distances of nearest neighbor pairs in the reconstructed state space. **(E)** Average logarithmic rate of divergence of all nearest neighbor pairs over time and the maximum Lyapunov exponent (λ_max_) as slope of the linear fit to the resulting divergence curve for 0 to 50 samples (0.5 s).

Local dynamic stability was examined using the maximum finite-time Lyapunov exponent (λ_max_) as a criterion for the assessment of the neuromuscular control of spine stability. Participants performed a lifting test, in which a pot (1.5 kg) was cyclically moved back and forth between two tables of different heights (90 and 53 cm; **Figure 2**). The tables were positioned forming an angle of 90° to each other, and the participant was standing in the middle of both tables in order to induce 45° of axial trunk rotation to each side. The positioning of the tables was standardized for all participants in order to ensure ecological validity, since usually situations in daily life are equal for all individuals independent of anthropometrical measures. The rhythm of 12 cycles/minute (0.20 Hz) was given by a metronome. Similar repetition rates (0.24–0.28 Hz) have been previously reported to be adequate for the assessment of local dynamic stability (Dupeyron et al., 2013; Graham et al., 2014). A total of 40 cycles were evaluated for the time series analysis. Three-dimensional kinematic data were collected using the Vicon 624 system (250 Hz) with 10 cameras. Markers (radius 14 mm) were placed on the anterior and posterior iliac spines and a marker triad (radius 6 mm) was placed at T12 (12th thoracic vertebrae). The 3D Euler rotation angles were calculated with the Yaw-Pitch-Roll sequence from the T12 coordinate system with respect to the hip, according to the ISB recommendation (Wu et al., 2002). For the non-linear time series analysis, we used the Euclidean norm of the three Euler angles resampled to 100 Hz. The reconstruction of the dynamic of the trunk motion in state space was performed using the method of delay embedding by choosing an appropriate time delay *τ* and embedding dimension *m* as follows:

(2) S​(t)=(s(t)​,s​(t+τ)​,s​(t+2τ)​,...,s​(t+(m−1)τ))

with *S*(*t*) being the *m*-dimensional reconstructed state vector, *s*(*t*) the one-dimensional Euclidean norm series, *τ* the time delay, and *m* the embedding dimension (**Figure 2**). The time delay was calculated individually with the first minimum of the average mutual information function (Fraser and Swinney, 1986), and the required reconstruction dimension was determined using global false nearest neighbor’s analysis (Kennel et al., 1992). The dimension of three was sufficient to reconstruct the state space in all the performed trials, and the temporal delay was 118.58 ± 9.92 time steps (following the down-sampling to a 100-Hz sample rate this corresponds to around 1.2 s). The maximum Lyapunov exponent as a measure of the local dynamic stability was then calculated using the algorithm of Kantz (1994) in the range 0–0.5 s. This parameter describes the average logarithmic divergence between initially neighboring trajectories in state space. Thus, the smaller the value of λ_max_ the more stable the system locally responds to small variations or perturbations.

### Statistical Analysis

The two-way analysis of variance and Bonferroni *post hoc* tests were used to examine the differences between groups (athlete and non-athlete) and LBP conditions for the muscle strength, onset times, stiffness, damping, and maximum Lyapunov exponent. The Shapiro–Wilk Test was performed to verify the normal distribution of the data and Levene’s test to assess the homogeneity of variances. The level of significance was set at α = 0.05.

## Results

No significant differences were present in the anthropometric data between the four groups of participants (**Table 1**). We found a significant pain effect on the maximum isometric trunk extension moments in all three investigated trunk positions, indicating a lower extension strength in LBP patients compared to healthy controls (*p* = 0.013–0.023; effect size η^2^ = 0.091–0.108) (**Table 2**). However, for the isometric trunk flexion, no pain-related significant differences were found in both athletes and non-athletes (*p* = 0.54–0.92). Athletes showed significantly (*p* = 0.001–0.009; effect size η^2^ = 0.132–0.186) higher maximal extension and flexion moments under both conditions (with and without LBP) compared to non-athletes (**Table 2**). We found no significant pain (*p* = 0.136) or group (*p* = 0.477) effects on trunk stiffness. Trunk damping was significantly higher for the LBP participants (*p* = 0.018; effect size η^2^ = 0.097), but did not show any group by LBP interaction (*p* = 0.331) indicating similar alterations in both athletes and non-athletes (**Table 3**). We also observed a significant pain effect on the muscle onset times of the lumbar (*p* = 0.019; effect size η^2^ = 0.073) and thoracic (*p* = 0.025; effect size η^2^ = 0.091) erector spinae muscles, evidencing shorter muscle reaction times after release in the LBP patients of both athlete and non-athlete groups (**Table 3**). No significant differences were found in the λ_max_ between groups or LBP conditions (*p* = 0.395 and *p* = 0.375, respectively; **Table 3**).

**Table 2:** Trunk maximal isometric flexion and extension moments normalized to body mass (Nm/kg) for the four groups of participants: elite athletes (athletes) with (LBP) and without (healthy) chronic non-specific low-back pain and participants of the general population (non-athletes), also with and without chronic non-specific low-back pain (means ± standard deviation).

Parameter	Non-athletes		Athletes	
Isometric contractions	Healthy (*n* = 14)	LBP (*n* = 15)	Healthy (*n* = 15)	LBP (*n* = 15)
Moment_Ext. -15°_ (Nm/kg)^#,∗^	3.65 ± 1.16	2.76 ± 1.03	4.20 ± 1.10	3.78 ± 0.84
Moment_Ext.5°_ (Nm/kg)^#,∗^	3.86 ± 1.08	3.37 ± 1.33	4.87 ± 0.44	4.10 ± 0.65
Moment_Ext.30°_ (Nm/kg)^#,∗^	4.17 ± 1.13	3.61 ± 1.28	5.19 ± 0.75	4.48 ± 0.92
Moment_Flex. -15°_ (Nm/kg)^#^	2.06 ± 0.43	1.81 ± 0.43	2.23 ± 0.34	2.34 ± 0.52
Moment_Flex.5°_ (Nm/kg)^#^	2.06 ± 0.37	1.85 ± 0.40	2.20 ± 0.27	2.38 ± 0.52
Moment_Flex.30°_ (Nm/kg)^#^	1.98 ± 0.31	1.83 ± 0.43	2.06 ± 0.32	2.26 ± 0.36

*^#^Significant (p < 0.05) group effect (athletes vs. non-athletes). ^∗^Significant (p < 0.05) low-back pain effect (healthy vs. LBP participants).*

**Table 3:** Trunk stiffness and damping coefficients normalized to body mass, onset times of the lumbar (OnTime_lumb_) and thoracic (OnTime_thorac_) erector spinae muscles after release and maximum Lyapunov exponent (λ_max_) for the four groups of participants: elite athletes (athletes), with (LBP) and without (healthy) chronic non-specific low-back pain and participants of the general population (non-athletes), also with and without chronic non-specific low-back pain (means ± standard deviation).

	Non-athletes		Athletes	
	Healthy (*n* = 14)	LBP (*n* = 15)	Healthy (*n* = 15)	LBP (*n* = 15)
Trunk stiffness coefficient [Nm/(rad^∗^kg)]	15.06 ± 5.63	11.51 ± 2.33	12.34 ± 2.60	12.73 ± 4.64
Trunk damping coefficient [Nm^∗^s/(rad^∗^kg)]^∗^	0.01 ± 0.47	0.32 ± 0.37	0.17 ± 0.31	0.36 ± 0.45
OnTime_lumb_ [ms]^∗^	79 ± 5	78 ± 9	81 ± 7	74 ± 5
OnTime_thorac_ [ms]^∗^	82 ± 8	77 ± 8	78 ± 2	73 ± 8
λ_max_	1.91 ± 0.33	1.69 ± 0.31	1.84 ± 0.33	1.90 ± 0.36

*^∗^Significant (p < 0.05) low-back pain effect (healthy vs. low-back pain participants) in both groups (athletes and non-athletes).*

## Discussion

In the current study, we aimed to investigate the athletic-based specificity of muscle strength and neuromuscular control of spine stability in non-specific LBP. Therefore, we compared the trunk muscle strength as well as the neuromuscular control of the spine after sudden quick release perturbations and during a repetitive lifting task in athletes and non-athletes with and without LBP. We hypothesized different pathology-related effects in athletes and non-athletes in trunk muscle strength and LBP related deterioration in neuromuscular control of spine stability in both groups. We found in athletes and non-athletes lower muscle strength of the trunk extensors during maximal isometric contractions and properly adapted neuromuscular spine control after the quick release perturbation in our LBP patients (i.e., shorter onset times of the erector spinae muscles and higher trunk damping after the quick release compared to healthy controls). These results indicate similar neuromuscular alterations in athletes and non-athletes, and therefore our hypothesis needs to be rejected. In agreement to earlier studies (Fenety and Kumar, 1992; Cho et al., 2014; Grosdent et al., 2015), we did not find any differences in the maximum trunk flexion moments between healthy and LBP participants in both the athlete and the non-athlete groups, indicating no specific LBP-related deterioration of the trunk flexor muscles.

Although chronic non-specific LBP is a complex and multifactorial process, a deconditioning of the lumbar extensor muscles has been often associated to chronic LBP (Rossi et al., 2015; Pienaar and Barnard, 2017). Furthermore, it is widely accepted that resistance training aiming to improve trunk muscles strength is a successful therapeutic modality for reducing LBP and improving functional outcomes (Jeffries et al., 2007; Steele et al., 2015). The average training volume of the athletes included in the study was 11 h per week with regular muscle strength exercising. Therefore, we expected at least a lower deconditioning of trunk extensor muscle strength compared to non-athletes. Yet we found a similar LBP-related decrease in the maximum trunk extension moments of up to 24% in both groups, indicating deficits in the trunk extensor muscle strength even at the high competitive level of athletes. A reason for this deficit could be the neglect of specific strength training focusing on the stabilization of the spine in athletes. Several review studies (Bolger et al., 2015; Jones et al., 2016) revealed that the majority of the practitioners recognize the benefits of strength training in athletes, but mainly focus on exercises to strengthen muscles which are directly related to the specific athletic performance, downgrading the importance of supplementary trunk stability or trunk strengthening exercises. Training recommendations for elite athletes mostly target superficial big groups of muscles (Kraemer et al., 1998) and less the deeper and smaller muscles, which stabilize the spine. Specific strength training for the trunk muscles seems not to be successfully integrated in the athletic practice compared to exercises, which target muscle groups that primary affect athletic performance. There are several reports providing evidence that strengthening the muscles of the lower extremities as for example plantar flexors, knee-hip extensors, and hamstring muscles provide important performance benefits in different sport-disciplines (Cristea et al., 2008; Cook et al., 2014; Keiner and Sander, 2014). On the contrary, a recent systematic review and meta-analysis study (Prieske et al., 2016) evidenced that trunk muscles strength training shows a limited association with athletic performance. However, our results indicate a deconditioning of the trunk muscles, not only in the general population but also in well-trained athletes with LBP. These results suggest that specific strength training of the trunk muscles could help patients to reduce LBP not only in the general population but also in a highly trained population like elite athletes. A reduction of LBP in the athletic practice would not only improve the health of athletes but would also have long-term beneficial effects on athletic performance (e.g., due to a reduction of pain-related absence from training sessions or competitions). Further prospective studies are needed to assess the effectiveness of specific conditioning therapies on LBP prevention in the athletic population.

Beside reduced muscle strength, deficits in the neuromuscular control of spine stability have been reported to be another possible risk factor for the occurrence of LBP. Especially after sudden perturbations, the ability of the nervous system to perceive sensory signals and generate appropriate motor commands stabilizing the spine can be a crucial element to protect the spine from injury and pain (Cholewicki et al., 2005; van Dieën et al., 2010). In our experiment, we did not find any differences in trunk stiffness between LBP and healthy participants neither in athletes nor in non-athletes, indicating an effective stabilization of the spine after the quick release perturbation. The challenge of stabilizing the trunk after the induced perturbation was quite high, and thus appropriate trunk stiffness was important to generate smaller and slower trunk displacement to counteract the perturbation. The LBP patients in both the athlete and the non-athlete groups showed a higher damping coefficient and shorter onset times of the erector spinae muscles (at the lumbar and thoracic level). An earlier activity of the trunk muscles in response to sudden perturbations may represent a strategy for pain and injury prevention (Cholewicki et al., 2005; Gildea et al., 2015). Damping is an important intrinsic factor in the musculoskeletal system and an essential component of spine stability control (Reeves et al., 2007). Higher damping values reflect an effective spinal control because a poorly damped trunk system would continue oscillating after a sudden perturbation (Reeves et al., 2007; Hodges et al., 2009). Therefore, an increased damping after sudden perturbations has been previously interpreted as beneficial for the effective control of spine stability in the presence of LBP (Hodges et al., 2009; Gildea et al., 2015). These findings indicate a properly adapted spine control after the quick release perturbation in our LBP patients. We can argue that the athletes and non-athletes with LBP included in our study did not present any deficits in the neuromuscular control of spine stability, at least compared to healthy controls.

The maximum Lyapunov exponents did not differ between the groups or the LBP conditions, indicating that the local dynamic stability of the trunk motion was independent of the presence of LBP in both athletes and non-athletes. Similar results (i.e., no LBP effects on local dynamic stability of trunk kinematics) were also reported by Graham et al. (2014) and Asgari et al. (2015). A recent study (Arampatzis et al., 2017) also found unchanged local dynamic stability of the trunk motion despite a significant reduction in LBP after an exercise therapy. To our knowledge, the only study that reported increased instability of trunk kinematics in the presence of LBP (Ross et al., 2015) used a heat-capsaicin model to introduce the LBP. It seems that a simulated acute increase in LBP may affect the ability of the spinal system to counteract and compensate neuromuscular control errors in order to maintain spine stability in a different manner as real chronic LBP in patients. It can be argued that our chronic non-specific LBP patients were able to overcome instabilities and control errors during the repetitive lifting task.

## Conclusion

We found similar LBP related consequences in elite athletes and in non-athletes for both muscle strength and neuromuscular control parameters of spine stability. Although the intensive and comprehensive training in competitive sports would be expected to differentiate the consequences and motor control adjustments of LBP, both athletes and non-athletes showed the same deconditioning of the lumbar extensor muscles and developed similar mechanisms to protect the spine from pain and damage. Further evaluation of specific training interventions to strengthen the trunk muscles is needed, not only for individuals of the general population but also for well-trained athletes. Body mass and BMI were not different between athletes and non-athletes. However, it might be possible that having the same BMI value athletes show a higher ratio muscle mass to fat affecting the normalization of muscle strength to body mass and the comparison between athletes and non-athletes. Nevertheless, our main finding was that in both athletes and non-athletes the LBP participants showed lower muscle strength values, and this result cannot be affected by the used normalization. Although general recommendations for MVC testing suggest the performing of more than one trial (Wyse et al., 1994), we decided to perform only one trial in each trunk position in order to be able to measure maximal strength in several angular positions and so assess for differences between groups in the force–length relationship. Our results showed similar differences between groups and pain condition in all the trunk positions used in our experimental design indicating stable results within the different trunk positions. We are therefore confident with our findings.

One limitation of this study was the lack of EMG recordings of the abdominal muscles. Studies from Cholewicki et al. (2002, 2005) using similar methods have shown the abdominal muscles shut-off latency to be a significant preexisting risk factor for the appearance of low-back injury. In the athlete population, individuals sustaining low-back injury showed latencies in average 14 ms longer than those who did not sustained any low-back injury in a prospective study (Cholewicki et al., 2005). For the assessment of muscle onset times, we minimized the possible methodological errors using cable EMG devices and using average values of five trials. Therefore, we can assume that the found significant differences were related to the sample characteristics and not to any methodological limitations. However, the small differences in the onset times between individuals with and without LBP, even if they reached Cohen’s criterion for medium effects (η^2^ = 0.091 for the thoracic and η^2^ = 0.073 for the lumbar muscles; Cohen, 1977), might not be big enough to be considered of clinical relevance.

Since the etiology of non-specific LBP is indeed multifactorial (Cholewicki et al., 2005), this pathology is not only related to neuromuscular deficits. Psychosocial factors like cognitive beliefs, emotional states, distress, or social context also play an important role related to the appearance and evolution of chronic LBP (Refshauge and Maher, 2006; Wippert et al., 2017). These factors were outside the scope of the current study and were not considered in the analysis.

## Data Availability Statements

Datasets are available on request. The raw data supporting the conclusions of this manuscript will be made available by the authors, without undue reservation, to any qualified researcher.

## Ethics Statement

This study was carried out in accordance with the recommendations of the Charité – Universitätsmedizin Berlin with written informed consent from all subjects. All subjects gave written informed consent in accordance with the Declaration of Helsinki. The protocol was approved by the ethics committee of the Charité – Universitätsmedizin Berlin.

## Author Contributions

AA, MMC, GL, and AS contributed to the conception and design of the study. MMC and GL planned and carried out the experiments. AS performed the numerical calculations for the suggested experiment. AA and MMC contributed to the interpretation of the results, wrote the manuscript, and critically revised the important intellectual content. All authors contributed to manuscript revision, read, and approved the submitted version.

## Conflict of Interest Statement

The authors declare that the research was conducted in the absence of any commercial or financial relationships that could be construed as a potential conflict of interest.
